# Antibiotic resistance by high-level intrinsic suppression of a frameshift mutation in an essential gene

**DOI:** 10.1073/pnas.1919390117

**Published:** 2020-01-28

**Authors:** Douglas L. Huseby, Gerrit Brandis, Lisa Praski Alzrigat, Diarmaid Hughes

**Affiliations:** ^a^Department of Medical Biochemistry and Microbiology, Biomedical Center, Uppsala University, Uppsala SE-751 23, Sweden

**Keywords:** rpoB, frameshift suppression, antibiotic resistance, evolution, gene regulation

## Abstract

A fundamental feature of life is that ribosomes read the genetic code in messenger RNA (mRNA) as triplets of nucleotides in a single reading frame. Mutations that shift the reading frame generally cause gene inactivation and in essential genes cause loss of viability. Here we report and characterize a +1-nt frameshift mutation, centrally located in *rpoB*, an essential gene encoding the beta-subunit of RNA polymerase. Mutant *Escherichia coli* carrying this mutation are viable and highly resistant to rifampicin. Genetic and proteomic experiments reveal a very high rate (5%) of spontaneous frameshift suppression occurring on a heptanucleotide sequence downstream of the mutation. Production of active protein is stimulated to 61–71% of wild-type level by a feedback mechanism increasing translation initiation. The phenomenon described here could have broad significance for predictions of phenotype from genotype. Several frameshift mutations have been reported in *rpoB* in rifampicin-resistant clinical isolates of *Mycobacterium tuberculosis* (Mtb). These mutations have never been experimentally validated, and no mechanisms of action have been proposed. This work shows that frameshift mutations in *rpoB* can be a mutational mechanism generating antibiotic resistance. Our analysis further suggests that genetic elements supporting productive frameshifting could rapidly evolve de novo, even in essential genes.

## Significance

Frameshift mutations have been reported in *rpoB*, an essential gene encoding the beta-subunit of RNA polymerase, in rifampicin-resistant clinical isolates of *Mycobacterium tuberculosis*. These have never been experimentally validated, and no mechanisms of action have been proposed. We show that *Escherichia coli* with a +1-nt frameshift mutation centrally located in *rpoB* is viable and highly resistant to rifampicin. Spontaneous frameshifting occurs at a high rate on a heptanucleotide sequence downstream of the mutation, with production of active protein increased to 61–71% of wild-type level by a feedback mechanism that increases translation initiation. Accordingly, apparently lethal mutations can be viable and cause clinically relevant phenotypes, a finding that has broad significance for predictions of phenotype from genotype.

Reading frame maintenance is essential for correct translation of the genetic code into protein (1). Frameshifting errors during translation are rare (10^−5^ to 10^−7^ per codon), and the effects of unplanned frameshifting are generally catastrophic for the resulting protein (2, 3). Error frequencies increase (typically 0.1–1%) on certain shift-prone sequences short enough to occur by chance (4), approaching 50% when a gene is very highly expressed off a multicopy plasmid (4–6).

In contrast, programmed ribosomal frameshifting (PRF) is promoted by evolved systems to direct the orderly slippage of ribosomes into a new reading frame at a specific site on messenger RNA (mRNA) during translation (7, 8). PRF is used to increase the information density of size-limited DNA sequences and serves regulatory roles in protein production (7). PRF generally requires the contribution of a pause site (to halt the progress of the ribosome), a slippery sequence (where the frameshift occurs), and a stimulator sequence that increases the frequency of frameshifting (7, 9, 10).

Considering the functional importance of reading frame maintenance during translation it is surprising that there have been published reports of frameshift mutations in the essential gene *rpoB* among rifampicin-resistant clinical isolates of *Mycobacterium tuberculosis* (Mtb) (11–14). To our knowledge none of these mutants have been investigated to determine either the validity of the mutation reported or a possible mechanism that could explain bacterial viability. The absence of investigation into this unexpected class of mutation may be due to the difficulty of performing complex genetic experiments in *M. tuberculosis*. There are several potential explanations for these observations. Leaving aside the trivial explanation (DNA sequencing errors) other possibilities include that the mutants carry a second functional copy of *rpoB* (unlikely if the DNA sequence analysis was done properly), that the mutants carry frameshift suppressor mutations (15–17), or that the mRNA contains sequence elements that promote a high level of ribosomal shifting into the correct reading frame to support cell viability (10). Interest in understanding these mutations goes beyond Mtb and concerns more generally the potential for rescue of mutants that acquire a frameshift mutation in any essential gene. We addressed this by isolating a mutant of *Escherichia coli* carrying a frameshift mutation in *rpoB* and experimentally dissecting its genotype and phenotypes.

## Results

### Isolation of a Frameshift Mutation in *rpoB* of *E. coli*.

**Fig. 1.**
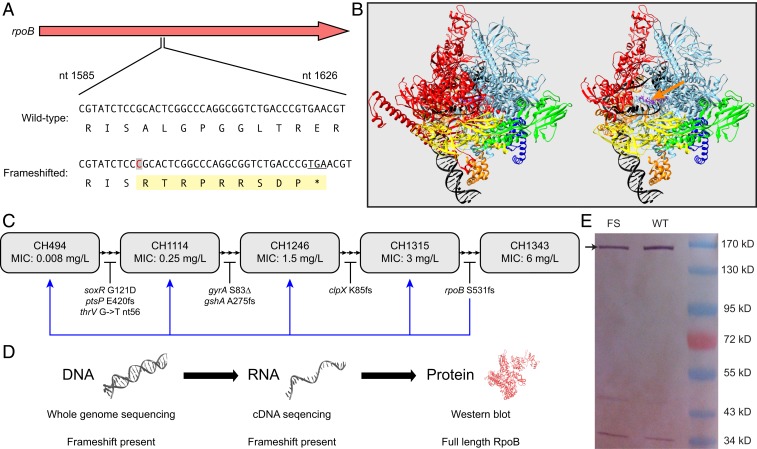
A frameshifted *rpoB* gene produces full-length RpoB protein without the need for a suppressor mutation. (*A*) Insertion of a single C nucleotide at position 1593 in *rpoB* that would be expected to result in mistranslated sequence of nine codons followed by a stop codon. In this scenario, the final 802 amino acids of RpoB would be untranslated. (*B*) Expected structural consequences of frameshift mutation visualized on RNA polymerase initiation complex (PDB 4YLN). The β subunit (RpoB) is shaded red, and the *Left* represents the full-length protein while the *Right* indicates the predicted consequence of the frameshift truncated protein. The exposed catalytic center is indicated by an orange arrow. (*C*) Evolutionary background in that the *rpoB* frameshift mutation was selected. Mutations were accumulated during selection as indicated by mutations below trajectory. MIC values indicate ciprofloxacin MIC at each sequenced step in the evolution. The blue arrow indicates that the *rpoB* frameshift mutation was transferred to each previous step in the evolution and was in all cases viable. (*D*) The presence of the frameshift mutation in genomic DNA and cDNA was confirmed by sequencing, but apparently full-length RpoB was observed by Western blot (*E*). Full-length RpoB has an expected mass of ∼160 kDa, while the frameshift truncated protein has an expected mass of ∼60 kDa.

Experimental evolution of ciprofloxacin resistance in *E. coli* frequently selects mutations in *rpoB* (18). During one such experiment a strain was isolated with a +1-nt insertion at codon Ser531 (TCC to TCCC) in the rifampicin-resistance–determining region (RRDR) of *rpoB* (Fig. 1). Sequencing of PCR-amplified DNA, and mRNA-derived cDNA (complementary DNA), confirmed the presence of the mutation (Fig. 1*D*). The mutation is predicted to result in a truncated RpoB protein, with Ser531 being followed by nine incorrect amino acids and a premature termination codon (Fig. 1*A*).

Since *rpoB* is essential, it is unlikely that a severely truncated form of the protein (19) would retain RNA-polymerase function (RpoB is 1,342 amino acid residues) (Fig. 1*B*). We reasoned that the frameshift mutation must be suppressed. To test this, Western blots were done using polyclonal rabbit antibodies targeted to the N terminus of RpoB (Fig. 1*E*). These showed that the mutant strain contained RpoB protein indistinguishable in length from that of wild-type *E. coli*. Visual examination of the Western blot suggested that the mutant contained at least 50% of the amount of RpoB present in wild-type *E. coli.* We concluded that the frameshift mutation is suppressed allowing production of full-length RpoB protein.

Since the mutation arose in an evolved strain containing additional mutations (Fig. 1*C*), we suspected that one of these might be a suppressor of the frameshift mutation. To test this, we transferred the *rpoB* mutation by phage P1-mediated transduction to strains with subsets of the mutations present in the final strain. The mutation was viable in all backgrounds, including in the wild-type parental *E. coli*. Whole-genome sequencing (WGS) confirmed that the mutant had not acquired a de novo suppressing mutation during transduction. We also reconstructed the mutation in a wild-type strain by recombineering. These experiments confirmed that a strain carrying the *rpoB* frameshift mutation in an otherwise wild-type background is viable. The relative growth rate of the mutant in Luria broth (LB medium) is 0.41 (wild type = 1). Because the mutation is in the RRDR we measured minimal inhibitory concentration (MIC) of rifampicin. MIC for the mutant is >1,500 mg/L (wild-type MIC = 12). Similar phenotypes (increased MIC and reduced growth rate) are associated with many rifampicin-resistance mutations caused by amino acid substitutions in RpoB (20).

### Identification of the Site of Frameshift Suppression.

Since no external mutation is required for suppression, we concluded the signal(s) allowing suppression of the frameshift were contained within the *rpoB* sequence itself. We noted that downstream of the site of the +1-nt insertion the codons that would be translated differed from those ordinarily observed in highly expressed genes like *rpoB*. Specifically, three codons downstream of the insertion, the di-codon CCC-AGG (Pro-Arg) was moved into the ribosomal reading frame by the mutation.

**Fig. 2.**
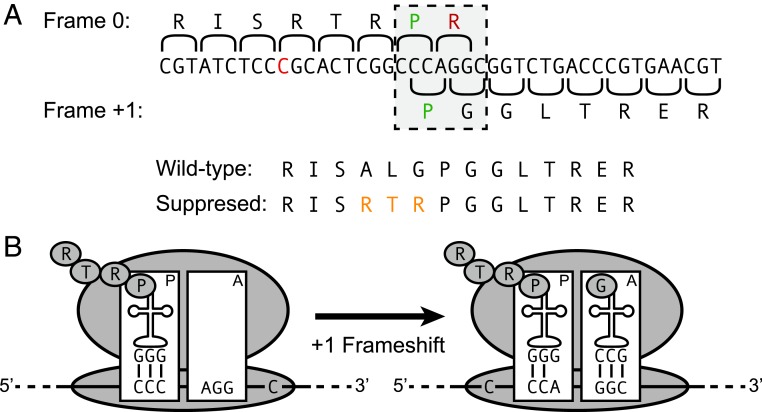
Model of the +1 frameshift to produce full-length RpoB protein. (*A*) Sequence of the *E. coli rpoB* gene in the vicinity of the frameshift site (red nucleotide), along with the predicted translation frame during frameshift suppression. The slippery proline is shown in green, and the rare arginine is shown in red. The protein sequence of the wild-type and suppressed protein products are shown below. Differences between the wild-type and suppressed sequence are indicated in yellow. (*B*) A Ribosome encountering the AGG codon must pause and wait for acylated tRNA^Arg^_CCU_ to arrive. Base-pairing between the P-site tRNA anticodon-codon transiently disassociates, and the mRNA slips in the ribosome, restoring the wild-type reading frame.

CCC is the least frequently used proline codon (21, 22) and is implicated in frameshifting as a “slippery” codon (16). AGG is the least frequently used codon in *E. coli* and never appears in highly expressed genes (*SI Appendix*, Table S1). This suggested a hypothesis for suppression of the frameshift mutation. The acylated transfer RNA (tRNA) pool of tRNA^Arg^_CCU_ (responsible for decoding AGG) is not optimized in tRNA level or acylation rate to satisfy the demands placed on it by the translation of this codon in highly expressed genes. Placing AGG into the reading frame of *rpoB* thus leads to depletion of acylated tRNA^Arg^_CCU_. Ribosomes encountering AGG must pause and wait for acylated tRNA^Arg^_CCU_ to arrive. The longer this pause, the greater the chance that base-pairing between the P-site tRNA anticodon-codon transiently disassociates and the mRNA slips in the ribosome, restoring the wild-type reading frame (23). This slippage event is potentiated by the presence of the slippery CCC codon in the P-site. The reason for the slippery characteristic of the CCC codon is that when an mRNA slips in the ribosome, the anticodon of the P-site proline tRNA can reestablish base-pairing with the sequence 1 nucleotide toward the 3′ end of the mRNA (CCN), particularly the important first- and second-position bases. The anticodon of the specific proline tRNA in the P site can affect the likelihood of a successful slippage event, as previously described (9). This hypothesis makes a specific prediction of the RpoB protein sequence that should be observed in the mutant if our hypothesis is correct (Fig. 2*A*).

To test this hypothesis, we assayed RpoB protein from wild-type and frameshift-bearing *E. coli* by LC-MS/MS (liquid chromatography with tandem mass spectrometry) analysis. We tested a variety of protein sequence hypotheses (*SI Appendix*, Fig. S1) extending upstream of the stop codon that was placed into the reading frame by the frameshift. From the wild type, the only fragments recovered corresponded to the wild-type sequence of RpoB (*SI Appendix*, Fig. S2 *A* and *B*). From the mutant, no wild-type fragments were recovered that spanned the site of the mutation, and the only protein sequence for which a fragment was recovered at the frameshift site corresponded to that predicted by our model, with a net substitution of ALG in the wild type by RTR in the mutant (Fig. 2 and *SI Appendix*, Fig. S2 *C* and *D*). This directly confirms that +1 frameshifting in the mutant occurs on the CCC-AGG-C sequence.

As a further test we constructed by recombineering a strain in which *rpoB* has the frameshift-suppressed sequence but without the frameshift (net replacement of amino acids 532–534, ALG to RTR). This strain has a relative growth rate of 0.78 (wild type = 1), confirming that the frameshifted RpoB protein supports viability.

### Quantitation of RpoB in the Mutant Frameshift Strain.

We quantified the amount of RpoB protein by LC-MS/MS, using two different assays. In one, specific target fragments in RpoB were chosen, calibration curves of these fragments were constructed by loading a range of masses of total protein from wild-type cells into the LC-MS/MS device, and the relative protein concentrations were determined by loading equal masses of wild-type or mutant proteins and comparing fragment recoveries. In the second, all fragments recovered from loading digested total protein were assigned to *E. coli* proteins, and relative amounts of each individual protein were calculated as a ratio of total cellular protein. The results of these two techniques were in good agreement with one another, with mutant RpoB protein levels being quantified as 59.6% (±21.1%) and 71.2% (±11.5%) of wild-type levels, respectively (*SI Appendix*, Tables S2 and S3).

The level of *rpoB* mRNA was assayed by qPCR to test whether transcriptional up-regulation contributed to the high level of RpoB protein in the mutant. We found similar levels of *rpoB* mRNA in mutant and wild-type cells (*SI Appendix*, Fig. S3). Accordingly, the level of RpoB protein from the frameshifted gene is solely due to factors acting on a translational level, such as frequency of frameshifting on the slippery site and/or up-regulation of translation initiation on *rpoB* mRNA.

### Determining the Minimal Sequence Required for Frameshifting in *rpoB*.

**Fig. 3.**
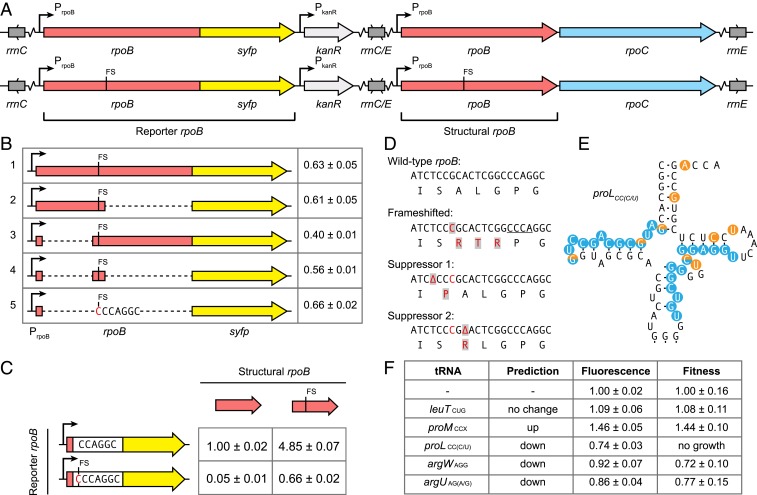
Identification of components required for high-level frameshift (FS) suppression. (*A*) Translational fusions of *rpoB* to the fluorescent reporter *syfp* were constructed in strains with trapped duplications of the *rrnC*-*rrnE* interval held with a kanamycin resistance cassette. (*B*) The minimal sequence requirements for high-level frameshift suppression were identified by deletions (dashed lines) of regions upstream and downstream of the site of the frameshift in the *rpoB-syfp* reporter strain. The first 100 amino acids of RpoB fused to 7 nt including the site of the frameshift insertion are sufficient for full-level suppression (line 5 vs. line 1). Values (mean ± SD, *n* = 3) were normalized to a strain containing the wild-type structural and reporter *rpoB* genes (*A*, *Top*). (*C*) Combinations of minimal *rpoB* fusions and structural *rpoB* genes reveal the contribution of frameshift suppression (top vs. bottom values) and translational up-regulation (left vs. right values) to *rpoB* expression. Values (mean ± SD, *n* = 3) were normalized to the strain containing the wild-type structural and reporter *rpoB* genes (*Top Left*). (*D*) Internal suppressors of slow growth phenotype of strains containing *rpoB* frameshift. (*E*) External suppressors of slow growth phenotype of strains containing *rpoB* frameshift isolated in the tRNA gene *proL* (total of 31 isolates). Orange circles represent nucleotides affected by mismatches, while blue circles indicate deleted or duplicated regions. All mutations are predicted to attenuate or abolish the function of the tRNA. (*F*) Overexpression of various tRNAs confirms model-based predictions of the efficiency of frameshift suppression. Fluorescence values (mean ± SD, *n* = 4) were measured in a strain containing a minimal *rpoB*-*syfp* fusion (7 nt) and a wild-type structural *rpoB* (*C*, *Bottom Left*). Growth measurements (mean ± SD, *n* = 9) were made in a strain containing the *rpoB* frameshift mutation. All measurements were normalized to the corresponding isogenic plasmid-free strain.

We asked whether there were sequence elements, in addition to CCC-AGG-C, that stimulate the frequency of frameshifting. We constructed *rpoB-syfp* translational fluorescent reporter fusions to address this. Since RpoB is an essential protein the fusions were constructed in strains carrying a trapped duplication of the *rpoB* region (Fig. 3*A*). *rpoB* is in a region of the chromosome flanked by ribosomal RNA (rRNA) operons that is frequently spontaneously duplicated (24). This duplication allowed us to make changes in one copy of *rpoB* while the unchanged copy in the secondary locus sustained viability. In all fusions the upstream regulatory region and first 100 codons of *rpoB* were kept intact to ensure wild-type regulation of the operon.

Through stepwise reductions in the length of the fusion leader sequence (Fig. 3*B*), we determined the minimal sequence required for frameshifting. A fusion containing CCC-AGG-C, frameshifted with respect to the *syfp* gene, expressed SYFP at levels similar to those observed for a strain carrying the frameshift mutation in the context of the complete *rpoB* sequence (Fig. 3*B*). This shows that the high level of frameshift suppression on CCC-AGG-C does not require any local upstream or downstream sequence elements.

### Determining the Level of Frameshifting on the Minimal Sequence.

We asked whether the high level of RpoB produced in the mutant was determined solely by the level of frameshifting on CCC-AGG-C or whether up-regulation of translation initiation on mRNA also contributed to the level of protein produced. RpoB production is known to be up-regulated at the translational level in response to RpoB starvation (25). We determined the level of frameshifting in a manner designed to isolate the effects of frameshift suppression from up-regulation at the translational level. We constructed minimal fusions with and without the frameshift mutation in two genetic backgrounds 1) with a wild-type *rpoB* gene in the secondary location expressing a wild-type level of RpoB and 2) with a frameshifted *rpoB* gene in the secondary location causing RpoB starvation (Fig. 3*C*).

The frameshifting rate on CCC-AGG-C was 5% in the wild-type *rpoB* background (Fig. 3*C*), which is not sufficient to explain the level of RpoB protein (60–71% of wild type) in a strain with the frameshift mutation (*SI Appendix*, Tables S2 and S3). However, translation of the *rpoB-syfp* fusions was increased 4.7-fold under RpoB-depleted conditions (Fig. 3*C*). We conclude that the amount of RpoB protein produced in *E. coli* with the frameshift mutation in *rpoB* results from a high-level frameshift (5%) at CCC-AGG-C, combined with a 4.9-fold increase of *rpoB* mRNA translation caused by RpoB starvation. The product of these two factors (24.5%) does not completely account for 66% frameshift suppression of *rpoB*-*syfp* in the background containing the frameshift mutation in the structural copy of RpoB (Fig. 3*C*). This discrepancy can be accounted for by the predicted increase in frameshifting under the burden of even greater translational demands on the pool of tRNA^Arg^_CCU_ caused by translational up-regulation of RpoB. The 66% frameshift suppression in this fusion strain closely matches the results of the direct protein quantitation (60–71% of wild type) (*SI Appendix*, Tables S2 and S3).

### Assessing the Importance of tRNA^Pro^ Slippage on the CCC Codon.

The CCC codon can be read by two different proline tRNA species: *proL* reads CCC/U while *proM* reads CCX (9, 26, 27). Our hypothesis is that tRNA^Pro^ reading CCC shifts onto CCA to mediate the frameshift. This predicts that the specific tRNA^Pro^ reading CCC should influence the frequency of shifting onto CCA when the ribosome is paused on AGG. It also predicts that increased reading of AGG should reduce the probability of frameshifting. We tested these predictions by 1) manipulating the levels of tRNAs predicted to increase or decrease frameshifting and 2) by analyzing mutants selected for increased frameshifting.

1) We cloned different tRNAs and expressed them in two different reporter strains (Fig. 3*F*). Increased reading of AGG reduced frameshifting (and frameshift-dependent growth rate), while increased reading of CCC by a tRNA that could shift to CCA increased frameshifting and frameshift-dependent growth rate (Fig. 3*F*) (28).
2) We evolved the *rpoB* frameshift strain to select mutations conferring a faster growth rate. Five clones assessed by WGS had acquired a mutation in *proL* while retaining the frameshift mutation in *rpoB* (Fig. 3*E*). Based on this we sequenced *proL* and *rpoB* (region surrounding the frameshift) from 33 independently evolved clones. Two clones had acquired −1-nt mutations near the +1-nt frameshift site in *rpoB*, restoring the correct reading frame with a net single amino acid substitution (S531P or A532R) (Fig. 3*D*). The remaining 31 clones retained the *rpoB* frameshift mutation but acquired mutations distributed throughout *proL* (14 different mutations including partial deletions) (Fig. 3*E*). Our interpretation is that mutations that reduce the activity of *proL* tRNA increase the likelihood of CCC being read by the “shifty” *proM* tRNA, increasing frequency of frameshifting (and frameshift-dependent growth rate).

These experiments support the hypothesis that frameshifting on CCC-AGG-C is initiated by ribosome pausing on AGG, increasing the probability that P-site tRNA (encoded by *proM*) shifts from CCC to CCA, placing the ribosome into a productive reading frame to make full-length RpoB.

## Discussion

Ribosomal frameshifting errors occur rarely, but when they do the consequences for protein function are generally catastrophic, and frameshift mutations are generally only observed in nonessential genes. In this context we were intrigued by reports of clinical isolates of Mtb carrying frameshift mutations (11–14), or nonsense mutations (12, 29–31), in the essential gene *rpoB*. Surprisingly, none of the publications reported any experimental analysis of the mutations or explained how these mutants were viable. Because frameshift mutations are not expected to be viable in this essential gene we set out to determine how such mutants could survive.

To address this, we isolated a frameshift mutation in *rpoB* in the experimentally amenable species, *E. coli*. We show here that an otherwise wild-type strain carrying a frameshift mutation in *rpoB* is viable, and the mutation generates a high level of resistance to the antibiotic rifampicin. We also detailed the mechanism of intrinsic frameshift suppression that supports bacterial viability. There are at least 120 different mutations, mostly amino acid substitutions but also deletions and additions of single or multiple amino acids tolerated in the RRDR, and causing resistance to rifampicin (20). This study expands this list of rifampicin-resistance mutations to include frameshift mutations in *rpoB*. Importantly, it shows that frameshift mutations, in the right sequence context, can generate viable selectable phenotypes in essential genes. This has significant implications in two additional fields: 1) the probability of de novo evolution of PRF systems and 2) the use of genome sequence analysis to predict phenotypes, in particular in clinical bacterial isolates.

The evolution of PRF systems in essential genes presents a conundrum. The first step in the evolution would necessarily be the frameshift, since associated stimulating sequences would have no selection by which to evolve in the absence of the need that the frameshift satisfies. The viability of a strain carrying this ostensibly lethal mutation suggests that PRF features may evolve by harnessing surprisingly high levels of spontaneous frameshifting under certain circumstances to then allow the evolution of more complex features to increase the frequency of frameshifting. This opens a path by which PRF can be evolved to serve productive roles, regulatory or otherwise.

The sequence on which +1 frameshifting occurs in the *rpoB* mutant (CCC-AGG-C) has similarities to the site of PRFs in yeast transposon TY1 (32) and the essential *E. coli* gene *prfB* encoding peptide release factor 2 (RF2) (33). In TY1 a +1 reading frame shift occurs at 20% efficiency on CUU-AGG-C (34). The ribosome pauses on the rare AGG codon, and during the pause leucine tRNA reading CUU shifts to read the cognate UUU codon, causing a frameshift into the +1 reading frame (32). The outcome in TY1 is production of two different proteins from the same genetic sequence (one short and one long). In *prfB* the +1-nt frameshift occurs with an efficiency of ∼50% on the in-frame CUU-UGA-C sequence. Ribosomes stall on the UGA stop codon, and leucine tRNA reading CUU shifts +1 onto the cognate UUU codon (35). The efficiency *prfB* frameshifting is stimulated by an upstream Shine-Delgarno sequence that base pairs with 16S rRNA (35), whereas the TY1 frameshift does not appear to require a stimulator sequence. The PRF in *prfB* functions to autoregulate RF2 production in response to demand. In these examples, and in the *rpoB* mutant, the common mechanism is ribosomal pausing on a starved or empty A-site codon, allowing a P-site tRNA to shift to an overlapping cognate codon resulting in the frameshift. The efficiency of intrinsic suppression of the *rpoB* mutation (5% frameshifting) suggests that the barrier to the de novo evolution of similar PRF systems is low.

Increasingly, WGS is used to predict phenotypes from genotypes. This is particularly interesting in clinical microbiology to predict antibiotic resistance and inform therapy. Genome sequence analysis is rapid and could provide significant diagnostic advantages over traditional phenotype-based methods with slow-growing organisms such as Mtb (36, 37). However, sequence analysis relies fundamentally on the assumption that ORFs accurately predict protein sequences. When frameshift (or nonsense) mutations are noted, the natural assumption will be made that they inactivate the gene in question (although this should raise a red flag if the gene is essential) and abolish production of the protein. Our results undermine this assumption, significantly complicating the process of predicting phenotype from genotype.

How to deal with this issue? Determine the sequence has been correctly called and that the mutation is not part of a PRF system characteristic of that species. If established as a genuine mutation, then assess its effect on product levels experimentally (proteomics analysis) and in silico, looking for similarities with known shifty sequences. With the exception of genes with well-known phenotypes (like *rpoB* and rifampicin resistance) there is currently no easy path to definitively predict the phenotypes of frameshift/nonsense mutations in most genes.

By examining publicly available genome sequences of 58 clinical Mtb isolates we found that each genome carried on average 68 (53–213) genes with frameshift mutations. These included mutations in essential genes such as *rpsK*, *rpsQ*, *infB*, *secA*, and *tilS* (*SI Appendix*, Table S4). This suggests that frameshift (and nonsense) mutations may be a hugely underappreciated class of mutations in clinical isolates, with largely unknown consequences for phenotype. The high frequency of such mutations in genome sequences strongly motivates the need to develop improved predictive genotype to phenotype methods.

In summary, we have elucidated how an apparent knockout mutation in *rpoB* is viable and causes antibiotic resistance. The wider implications are that there is considerable potential for de novo evolution of new PRF systems in bacterial genomes, and there is a need to develop sophisticated approaches to predict phenotype from genome sequence data.

## Materials and Methods

### Experimental Evolution.

Cultures of a media-adapted wild-type *E. coli* MG1655 were grown from independent single colonies overnight at 37 °C in Mueller–Hinton (MH) broth (Becton-Dickson). From each culture, 100-µL aliquots were spread onto five MH agar plates containing different ciprofloxacin concentrations, 0.032, 0.064, 0.096, 0.128, and 0.16 µg/mL, corresponding to 2, 4, 6, 8, and 10 times the MIC of the parental strain. Plates were incubated for up to 48 h and examined for the growth of colonies. From each culture a single colony was picked from the highest drug concentration where growth occurred (defined as colony diameter ≥1 mm within 48 h). Each colony was purified by streaking on MH agar plates with the drug concentration at which it was isolated. Second-step mutants were selected by resuspending a colony of each of the purified first-step mutants in 0.9% NaCl and plating ∼10^8^ colony-forming units onto MH agar plates with ciprofloxacin at 2, 4, 6, 8, and 10 times the concentration at which the mutant was originally selected and purified. Selection was continued through 3–8 successive cycles until the concentration at which mutants were being selected reached or exceeded 1 mg/L.

### Whole-Genome Sequencing.

Genomic DNA was prepared using Genomic-tip 100/G kit and Genomic DNA Buffer Set (Qiagen), according to the instructions of the manufacturer. Genomic DNA was sent to BGI for library assembly and genome sequencing with Illumina sequencing technology. Sequencing data were aligned and analyzed using CLC Genomic Workbench v6 (CLC Bio).

### Local Sequencing of PCR Products and cDNA.

Total RNA was purified from exponentially growing cultures using RNeasy Mini Kit (Qiagen). RNA was reverse-transcribed using High Capacity cDNA Reverse Transcription Kit (Applied Biosystems, Thermo Fisher). PCR amplification for local sequencing of genomic DNA and cDNA was done using PCR Master Mix (Thermo Scientific) according to manufacturer specifications. DNA sequencing of PCR products was performed at Macrogen Europe. Sequences were analyzed using CLC Main Workbench 7.

### Genetic Methods.

The *rpoB* mutation was moved by P1-mediated transduction (38) into strains that contained subsets of the mutations present in the final evolved strain, with selection for a linked chloramphenicol-resistance marker, *yjaG*::*cat-sacB*. Lambda Red recombineering was used to generate a variety of genetic constructions (39). The frameshift mutation was directly reconstructed in wild-type *E. coli* MG1655 by transformation with an 81-mer oligonucleotide containing the mutation flanked by 40 nucleotides of *rpoB* homology on either side and selecting for slow-growing transformants on LB agar plates containing rifampicin at 100 µg/mL. Fusions of SYFP to various lengths of RpoB were constructed by designing oligonucleotides that would replace the 3′ end of *rpoB* with SYFP and a *kanR* cassette (40). The SYFP gene was translationally fused to the remaining fragment of *rpoB*, while the *kanR* gene retained its own native promoter. The deletion of the C-terminal region of RpoB in these constructions assured that any kanamycin-resistant colonies had duplicated the *rpoB* region; subsequent whole-genome sequencing of the resultant strains revealed that the trapped duplication was invariably between *rrnC* and *rrnE*, a duplication of roughly 260 kb.

### Growth Rate and Fluorescence Measurements.

Cultures were started by diluting a stationary-phase overnight culture 1:1,000 in fresh LB media. Growth rates were measured using a Bioscreen C Device (Oy Growth Curves) incubating at 37 °C with shaking. Readings of OD600 were taken every 4 min, and doubling times were calculated using the first 10 readings after the OD600 value exceeded 0.015. Fluorescence values were measured in a MACSQuant VYB device (Miltenyi Biotech) using exponentially growing cells collected at OD600 of 0.2–0.3. For each replicate the fluorescence of 100,000 single-cell events was measured.

### Protein Analysis.

Protein gels, Western blotting, protein sequencing, and quantification by two different methods were performed as described in *SI Appendix*, *Supplementary Materials and Methods*.

### Data Availability Statement.

All data discussed in the paper are available in the main text and *SI Appendix*. The paired-end sequence reads of the evolved strain carrying the frameshift mutation in *rpoB* (strain CH1343) and the reconstructed wild-type strain carrying the frameshift mutation (strain HS297) have been deposited to the NCBI Bioproject database with accession number PRJNA601628.

## Supplementary Material

Supplementary File

Supplementary File

Supplementary File

Supplementary File

Supplementary File
