# Supplementary material for: Antibiotic resistance by high-level intrinsic suppression of a frameshift mutation in an essential gene
Source: Proc Natl Acad Sci U S A. 2020 Jan 28;117(6):3185–91. doi: 10.1073/pnas.1919390117 (PMC7022156; doi:10.1073/pnas.1919390117)
Supplement: Supplementary File [file pnas.1919390117.sapp.pdf]

PNAS
www.pnas.org

Supplementary Information for

Antibiotic resistance by high-level intrinsic suppression of a frameshift mutation in an essential gene

Douglas L. Huseby, Gerrit Brandis, Lisa Praski Alzrigat, Diarmaid Hughes

Diarmaid Hughes
Email: diarmaid.hughes@imbim.uu.se

This PDF file includes:

Figures S1 to S3

Supplementary Materials and Methods

Other supplementary materials for this manuscript include the following:

Datasets Tables S1 to S4 (Excel files)

Fig. S1. Alternative protein sequences tested in LC-MS/MS protein sequencing.

Wild-type RpoB sequence	LSEITHKRRISALGPGGLTRERAGFEVRDVHPTHYGRVCP
Primary sequence prediction	LSEITHKRRISRTRPGGLTRERAGFEVRDVHPTHYGRVCP
Alternative prediction 1	LSEITHKRRISRTRPRRSDPERAGFEVRDVHPTHYGRVCP
Alternative prediction 2	LSEITHKRRISRTRPRRSDRERAGFEVRDVHPTHYGRVCP
Alternative prediction 3	LSEITHKRRISRTRPRRSTRERAGFEVRDVHPTHYGRVCP
Alternative prediction 4	LSEITHKRRISRTRPRRLTRERAGFEVRDVHPTHYGRVCP
Alternative prediction 5	LSEITHKRRISRTRPRGLTRERAGFEVRDVHPTHYGRVCP
Alternative prediction 6	LSEITHKRRISRTGPGGLTRERAGFEVRDVHPTHYGRVCP
Alternative prediction 7	LSEITHKRRISRLGPGGLTRERAGFEVRDVHPTHYGRVCP

Figure S1. *Alternate protein sequences tested in LC-MS/MS protein sequencing. Fragments recovered in LC-MS/MS analysis of RpoB protein from wild-type and frameshift-containing E. coli were tested against hypothetical protein sequences. Each hypothetical sequence represents a different amino acid at which the ribosome could have slipped back into the wild-type reading frame. The highlighted amino acids indicate those which would be different from wild-type in each hypothetical protein. Each of the peptide fragments depicted begins at amino acid 521 in the RpoB sequence, but in all cases the full-length modified RpoB protein was used as the query in the analysis.*

Figure 2

Figure S2*A*.

Wild-type *E. coli* RpoB chymotryptic digest tested versus wild-type RpoB sequence hypothesis

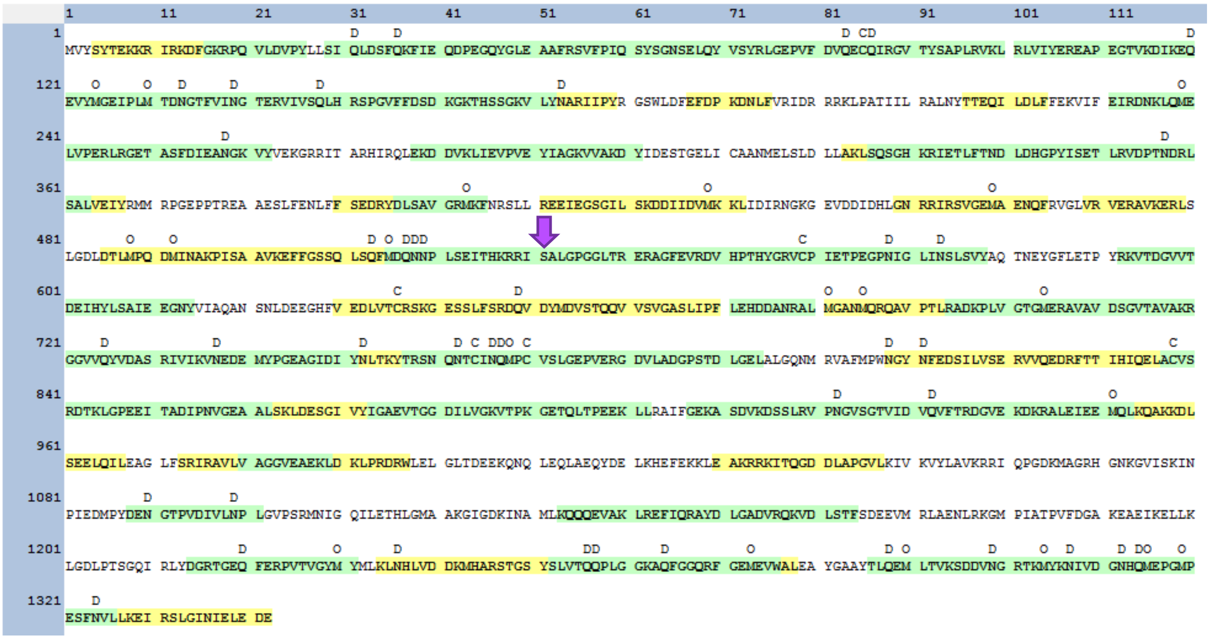

Figure S2*B*.

Wild-type *E. coli* RpoB chymotryptic digest tested versus primary RpoB frameshift suppression hypothesis

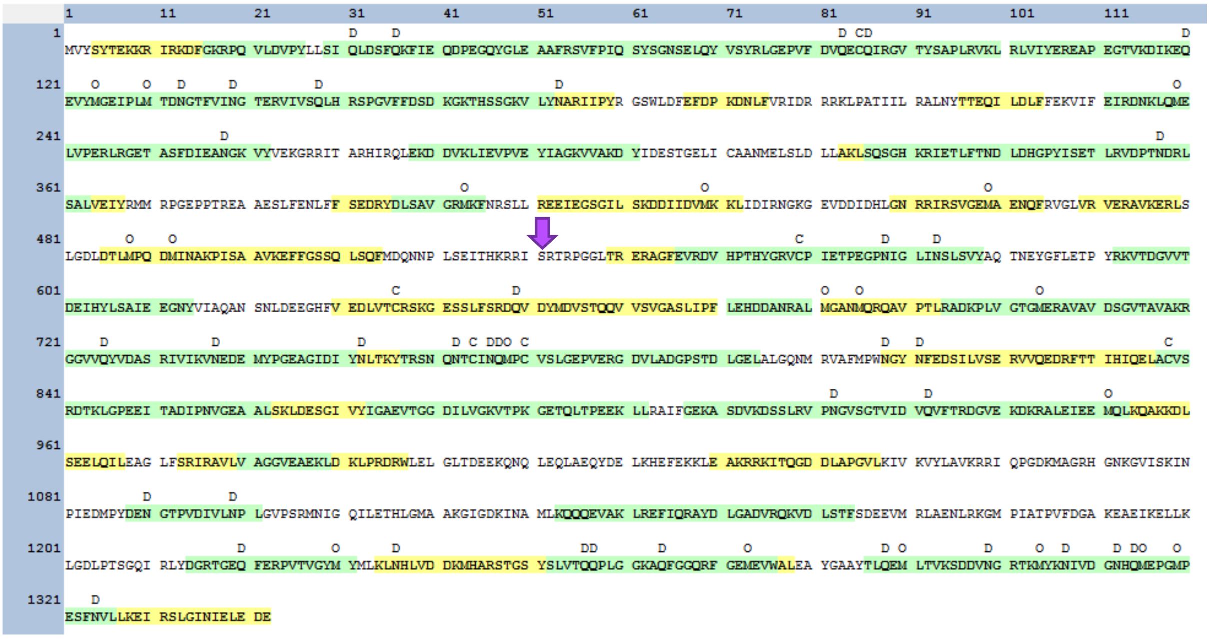

Figure S2*C*.
Frameshifted RpoB mutant chymotryptic digest tested versus wild-type RpoB sequence hypothesis

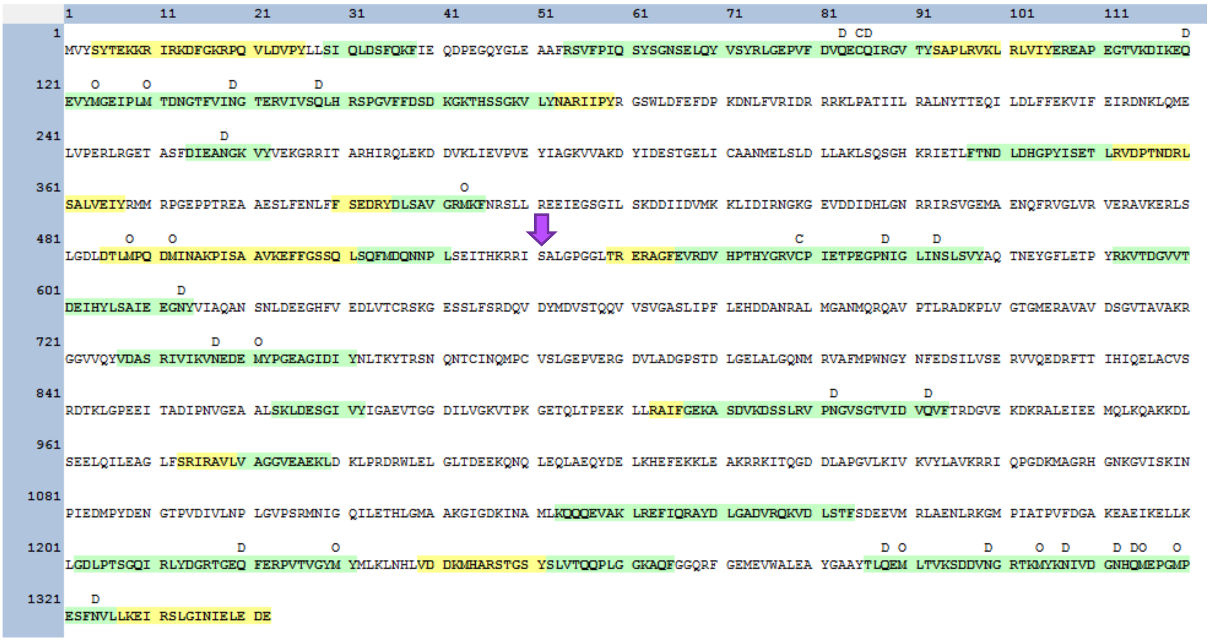

Figure S2*D*.
Frameshifted RpoB mutant chymotryptic digest tested versus primary RpoB frameshift suppression hypothesis

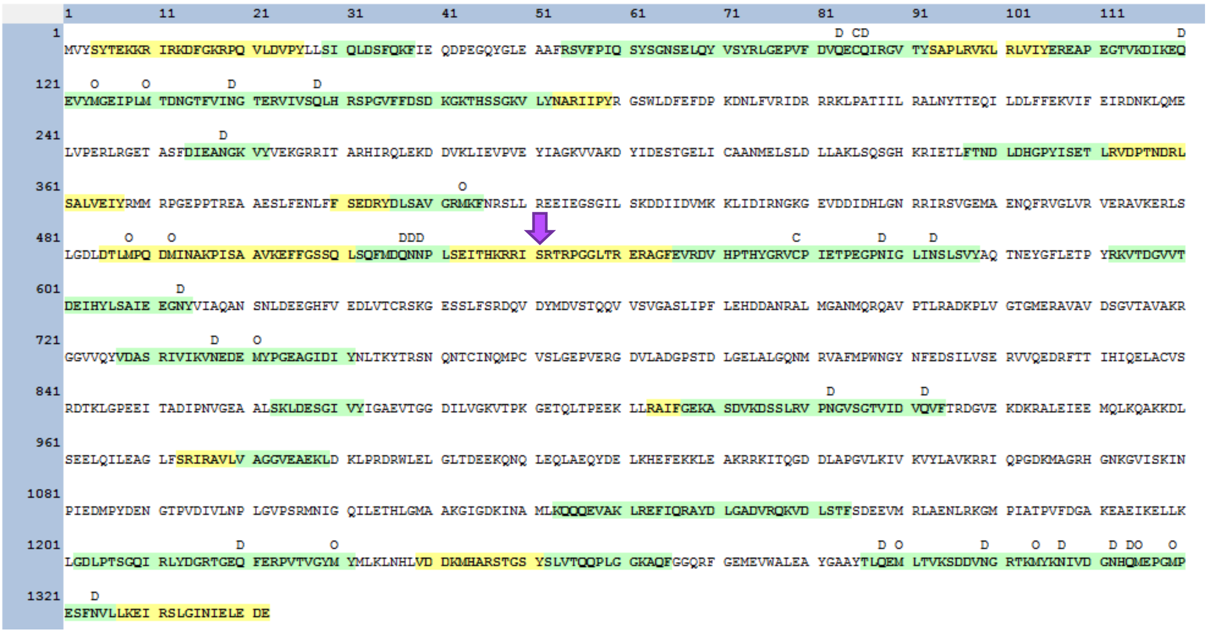

Figure S2. *Protein LC-MS/MS results from wild-type and mutant RpoB proteins. Highlighted sequences indicate observed fragments, with yellow highlighting indicating 95% significance and green highlighting indicating 99% significance. Purple arrows indicate the site of the frameshift insertion in the mutants and consequently the location where any amino acid changes caused by the frameshift mutation would begin in the protein sequence.*

Figure S3
qPCR to assess copy number of rpoB mRNA.

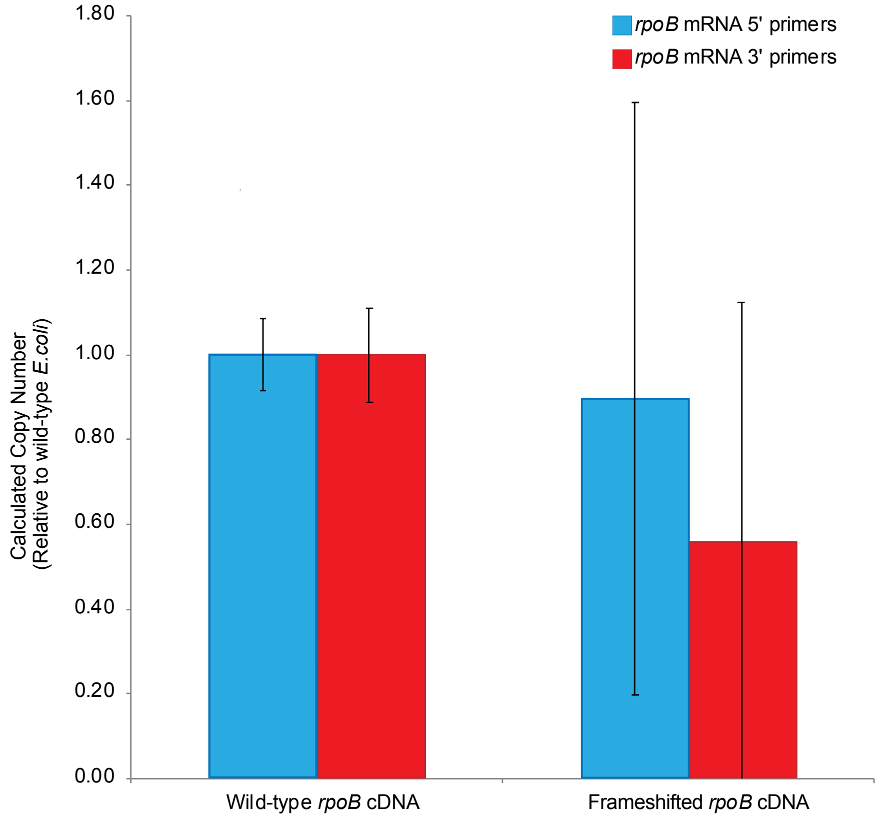

Figure S3. *qPCR to assess copy number of rpoB mRNA. qPCR using probes that target sequences upstream and downstream of the site of the frameshift in rpoB on cDNA derived from cellular mRNA. Expression was normalized using cysG, idnT and hcaT as internal controls (1). Error bars represent SD. Reduced copy number downstream of the frameshift mutation may represent degradation of messages unprotected by translating ribosomes. High error values in frameshifted strains may be a result of frequent duplication/amplification of rpoB genomic region under RpoB protein limitation within clones and cultures.*

Supplementary Materials and Methods.

Protein Gels and Western Blotting

Cultures of *E. coli* were grown to exponential growth phase in LB (OD600 = 0.3) and 2 mL of each culture was pelleted in a microfuge and frozen for subsequent analysis. The frozen pellets were resuspended in 50 µL of SDS-PAGE loading buffer and heated to 95°C for 10 minutes. Samples were once again pelleted at maximum speed in a microfuge for 5 minutes. 10 µL of the supernatant was loaded in duplicate onto a 7.5% Mini-Protean TGX Gel (Bio-Rad, CA, USA) and run for 30 minutes at 250 V in a Mini-Protean Tetra System gel apparatus (Bio-Rad, CA, USA). For Western blotting, proteins were transferred to Hybond ECL nitrocelluose membrane (Amersham Biosciences, Germany) in buffer containing 25 mM Tris, 192 mM glycine, and 10% methanol using a Trans-blot Cell apparatus (Bio-Rad, CA, USA) running at 50 V for 30 minutes at 4°C with buffer circulation. The membrane was blocked with gelatin. The primary antibody used in the blotting procedure was a 1:100 dilution of custom rabbit polyclonal antibody recognizing two peptides in the N-terminal region of RpoB (peptide sequences TEKKRIRKDFGKRPQ and FFDSDKGKTHSSGKV) (Eurogentec, Belgium). The secondary antibody used was a goat anti-rabbit alkaline phosphatase conjugate (Bio-Rad, CA, USA) at a 1:3000 dilution. Colorimetric development of the membrane was done using AP Conjugate Substrate Kit (Bio-Rad, CA, USA).

***Protein Sequencing by* LC-MS/MS.**

RpoB protein bands, migrating at approximately 150 kD, were cut from Coomassie-stained SDS-PAGE gels. The proteins were reduced, alkylated and in-gel digested either by trypsin or chymotrypsin according to a standard operating protocol. The samples were dried and resolved in 15 µL 0.1% formic acid. The peptides were separated in reversed-phase on a C18-column and electrosprayed on-line to an LTQ-Orbitrap Velos Pro ETD mass spectrometer (Thermo Finnigan, CA, USA). Tandem mass spectrometry was performed applying CID. Peptide mapping was performed towards the sequence of the wild-type protein and the hypothetical mutant protein sequences. Database searches were performed in MASCOT (Matrix Science, UK) towards proteins from all bacteria in the Swiss-Prot database, to check the quality of the samples.

LC-MS/MS Protein Quantitation Methods 1

Quantitative protein sequencing was performed at the Uppsala University MS-platform according to the following protocol. The total protein concentration in the samples was measured using the Bradford Protein Assay with bovine serum albumin (BSA) as standard. Aliquots corresponding to 20 g protein were taken out. These were reduced, alkylated and in-solution digested by trypsin according to a standard operating protocol. Thereafter the samples were purified by ZipTips® (Millipore, MA, USA), dried and resolved in 0.1% FA. Samples were selected for a qualitative analysis of the proteins in the samples. The peptides were separated in reversed-phase on a C18-column and electrosprayed on-line to an LTQ-Orbitrap Velos Pro ETD mass spectrometer (Thermo Finnigan, CA, USA). Tandem mass spectrometry was performed applying CID. For the quantitative analysis the mass spectrometer was operating in alternating SIM- and tandem mass spectrometry mode. The SIM windows were defined between 200 and 250 amu over the mass range m/z 400 – 1250. Targeted MS/MS, applying collision-induced dissociation, was performed using an inclusion list with 3 – 12 specified peptides for each target protein. The inclusion list was developed using the Pinpoint 1.3 software (Thermo Scientific, MA, USA). A dilution series with five concentrations of one sample was run for evaluation of the linear response in the mass spectrometer. For identification of proteins in the qualitative analysis of samples, database searches were performed in Sequest towards proteins in a FASTA database containing proteins from *E. coli* (strain K12). The search criterion for protein identification was set to at least two peptides of 95% confidence per protein. To assure correct peptide identity via reliable MS/MS spectra in the quantitative analysis, database searches of all samples analysed with the targeted method were performed as described for the qualitative analysis. These results were used for final selection of peptides (2 – 3 per protein) to be included in the final quantitation. Proteins without reliable MS/MS spectra were excluded. The quantitation analysis was performed in the Pinpoint 1.3 software, using the peak area of the precursor ion for each peptide. A calibration curve for each peptide was constructed from the dilution series of the calibration sample. This was thereafter used to convert the peak areas of the peptides into concentration in arbitrary units. These values were used to compare protein abundance of a specific protein between the bacterial strains. Peptide abundances were strongly affected by the position in RpoB in strains containing the frameshift mutation. Fragments upstream of the frameshift mutation were present at abundances similar to the wild-type levels, while those downstream of the mutation were depleted. Since our interest was in the abundance of full-length RpoB, only fragments downstream of the frameshift mutation were used to calculate abundances.

LC-MS/MS Protein Quantitation Method 2

Relative quantification of peptides was performed by the Proteomics Core Facility at Sahlgrenska Academy, Gothenburg University. Samples were homogenized in 200 µl lysis buffer (50 mM Triethylammoinium bicarbonate (TEAB; Fluka, Sigma Aldrich) and 8M Urea using a FastPrep®-24 instrument (MP Biomedicals, OH, USA). The sample buffer was adjusted to 2% SDS, put on a shaker for 30 min and total protein concentration was determined with Pierce™ BCA Protein Assay (Thermo Scientific, MA, USA). The same amount of total protein was analyzed for each sample. The proteins were trypsin digested using the filter-aided sample preparation (FASP) method (2) and peptides subjected to chemical labelling using isobaric mass tagging reagent TMT® according to the manufacturer's instructions (Thermo Scientific, MA, USA). The labelled samples were mixed per TMT set, fractionated by strong cation exchange (SCX) and desalted before LC-MS/MS analysis on a Q Exactive hybrid instrument (Thermo Fisher Scientific, MA, USA) interfaced with an Easy-nLC autosampler (Thermo Fisher Scientific, MA, USA). Peptides were separated on-line with a 0.075 mm inner diameter C18 reversed phase column using 0.2% formic acid and an acetonitrile gradient. Ions were injected in data-dependent positive ion mode and MS/MS spectra were acquired using higher energy collision dissociation (HCD) in a stepped format at 25, 35, and 45% from m/z 110 for the ten most abundant precursors at a resolution of 35,000. Dynamic exclusion during 30 s after selection for MS/MS was enabled to allow for detection of as many precursors as possible. Data analysis for relative quantification and identification was performed with Proteome Discoverer version 1.4 (Thermo Fisher Scientific, MA, USA). A database search was performed against Uniprot *E. coli* (strain K12), MS peptide tolerance of 5 ppm and MS/MS tolerance for identification of 50 millimass units (mmu). Tryptic peptides were accepted with zero missed cleavages and the threshold in the software was set to 1% False Discovery Rate by searching against a reversed database. The relative quantification was calculated for fragment ions within 3 mmu and co-isolation of less than 10% was allowed. Only peptides unique for a given protein were considered for relative quantification and ratios normalized using the protein median. As described for Method 1, only fragments downstream of the site of the frameshift were used to calculate the relative abundance of full-length RpoB protein.

Dataset Tables S1 – S4 (separate files).

Table S1. Codon Usage Table

Table S2. RpoB Protein Sequencing Method 1

Table S3. RpoB Protein Sequencing Method 2

Table S4. Mtb Frameshift Data

References

1. K. Zhou *et al.*, Novel reference genes for quantifying transcriptional responses of Escherichia coli to protein overexpression by quantitative PCR. *BMC Mol Biol* **12**, 18 (2011).
2. J. R. Wisniewski, A. Zougman, N. Nagaraj, M. Mann, Universal sample preparation method for proteome analysis. *Nature methods* **6**, 359-362 (2009).
